# Draft Genome Sequence of *Streptomyces ahygroscopicus* subsp. *wuyiensis* CK-15, Isolated from Soil in Fujian Province, China

**DOI:** 10.1128/genomeA.01125-15

**Published:** 2015-10-08

**Authors:** Beibei Ge, Yan Liu, Binghua Liu, Kecheng Zhang

**Affiliations:** State Key Laboratory for Biology of Plant Diseases and Insect Pests, Institute of Plant Protection, Chinese Academy of Agricultural Sciences, Beijing, China

## Abstract

We report the first high-quality draft genome sequence of an antibiotic (wuyiencin)-producing strain, *Streptomyces ahygroscopicus* subsp. *wuyiensis* CK-15, isolated from soil samples collected from Fujian Province, China. The 9.41-Mb genome comprises 8,311 protein-coding sequences, encodes 89 structural RNAs, and shows a G+C content of 72.25%.

## GENOME ANNOUNCEMENT

*Streptomyces ahygroscopicus* subsp. *wuyiensis* CK-15, a Gram-positive filamentous bacterium isolated from a soil sample collected from the Wuyi Mountains in Fujian Province, China, is of interest, as it expresses the antibiotic wuyiencin, which has wide applications as an agricultural antibiotic to control various fungal diseases of vegetable and field crops (1). This strain has been preserved at the China General Microbiological Culture Collection Center (CGMCC no. 0730) and can be cultured at 28°C on mannitol-soybean (MS) agar or in a yeast extract-malt extract (YEME) liquid medium (2). The soluble fermentation medium comprises the following (per 100 mL): 4 g soybean flour, 2 g glucose, 3 g corn starch, 300 mg CaCO_3_, and 100 mg (NH_4_)_2_SO_4_.

Genome sequencing was performed on both the Illumina MiSeq platform (Illumina, Inc.), by generating a paired-end library with an insert size of about 500 bp, and on the Illumina HiSeq2000 platform (Illumina, Inc.), by generating a mate-pair library with an insert size of about 5 kb; sequencing was performed at Sangon Biotech Co., Ltd. (Shanghai, China). Paired-end reads were *de novo* assembled using Velvet version 1.2.07 (3), and GapCloser version 1.12 (4) was used to fill the gaps. Gene prediction was performed using the RAST annotation server (5). Gene functional annotation was based on a BLASTp comparison with the nonredundant (NR), Kyoto Encyclopedia of Genes and Genomes (KEGG) (6), Protein Families (PFAM) (7), and Clusters of Orthologous Groups (COG) databases (8).

The genome size of *S. ahygroscopicus* subsp. *wuyiensis* CK-15 was 9,410,232 bp, with a 72.25% G+C content, which is similar to the 72.2% G+C content of the *Streptomyces griseus* subsp. *griseus* NBRC 13350 (NC_010572.1). A total of 130 contigs were obtained. Analysis of the genome revealed that it contained 8,311 protein-coding genes (CDSs), which accounted for approximately 83.38% of the genome. Among the 8,311 open reading frames (ORFs), clear functions could be identified for about 7,914 (95.22%) ORFs, while no match could be found for the remaining 397 (4.78%) using the NR protein database. In addition, the genome encoded 89 structural RNAs, including 7 5S rRNAs, 6 16S rRNAs, 8 23S rRNAs, and 68 tRNAs.

The antibiotic wuyiencin synthesized by the CK-15 strain shows a high similarity with an antibiotic produced by *Streptomyces nourser* ATCC 11455 (nystatin). The DNA sequence analysis of the gene clusters related to antibiotic biosynthesis (including *nysDI*, *nysDII*, *nysDIII*, *nysA*, *nysE*, *nysF*, *nysG*, *nysH*, *nysM*, *nysN*, *nysRI*, ORF2, and ORF3) revealed at least an 80% sequence homology between the CK-15 and ATCC 11455 strains. Further analyses revealed that the function of certain genes was similar; for example, ORF2 in both strains enhanced antibiotic production (2). This draft genome sequence will assist in developing a deeper understanding of the regulatory mechanisms underlying antibiotic production, thereby allowing the construction of high-yield wuyiencin strains for large-scale industrial production.

### Nucleotide sequence accession numbers.

The draft genome sequence of *Streptomyces ahygroscopicus* subsp. *wuyiensis* CK-15 has been deposited at DDBJ/EMBL/GenBank under the accession number JXYI00000000. The version described in this paper is the second version, JXYI02000000.
